# Acetabular fracture after cycling related falls: High index of suspicion is required to avoid missing the injury on plain radiographs

**DOI:** 10.17159/2078-516X/2022/v34i1a14526

**Published:** 2022-01-01

**Authors:** J Swart, M Horak, R de Villiers, C Oberholzer, A Rotunno

**Affiliations:** 1 HPALS Research Centre. Department of Human Biology, University of Cape Town, Sports Science institute of South Africa; 2 University of Stellenbosch, Faculty of Health Sciences, Stellenbosch, South Africa; 3 Sports Science Radiology practice, Winelands Radiology. Newlands, Cape Town, South Africa; 4 Carla Oberholzer Physiotherapy, Medical Centre, Main Street, Clarens, South Africa; 5 Cape Sports Medicine, Sports Science Institute of South Africa, Newlands, Cape Town, South Africa

**Keywords:** cycling, traumatic injuries, epidemiology

## Abstract

Cycling participation as a medium of transport and as a competitive sport has steadily increased in recent decades. Traumatic injuries secondary to falls and collisions occur relatively frequently. Fractures of the hip and pelvis are uncommon with no studies to date reporting their exact incidence in this sport. Injuries specific to the acetabulum are reported even less frequently. We present four cases that highlight the insidious nature of acetabular fractures in cyclists and document their management and recovery. The number of acetabular fractures following falls from bicycles directly onto the lateral hip result in a relatively high number of fractures. Many of these may be missed due to the absence of findings on plain x-ray imaging.It is therefore important to have a high index of suspicion for hip and pelvis fractures when treating cycling related traumatic injuries.

## Case report

Cycling as a medium of transport and as a competitive sport has steadily increased in recent decades.^[1,2]^ Traumatic injuries secondary to falls and collisions occur relatively frequently. The majority of these injuries are abrasions and contusions with fractures of the hand, wrist and clavicle the more common bony injuries. Fractures of the ankle and lower leg also occur relatively frequently.^[3,4]^

In comparison, fractures of the hip and pelvis are less common, with no studies to date reporting their exact incidence in this sport. Injuries to the acetabulum are reported even less frequently and only two case reports with respect to cycling-related acetabular injuries have been published to date.^[5,6]^

There have been a concerning number of accounts suggesting that habitual road cycling may predispose one to low bone mineral density in the hip and lumbar spine.^[7,8]^ It is therefore important to have a high index of suspicion for hip and pelvis fractures when treating cycling-related traumatic injuries.

We present four cases that highlight the insidious nature of acetabular fractures in cyclists and document their management and recovery.

### Case 1

**Fig. 1:**
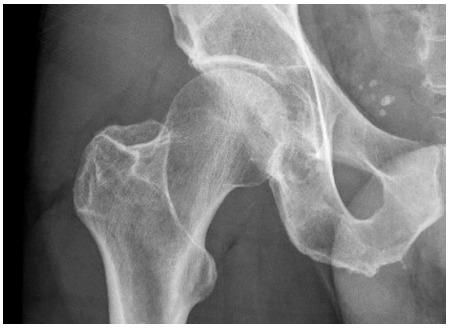
Case 1: X-ray of the right hip on the day of injury

A 64-year-old competitive cyclist sustained a fall while traversing technical off-road trails while riding his mountain bike. He fell directly onto his right side, predominantly the hip and flank. He was able to continue cycling but developed progressive pain while bearing weight on the right side after completing his cycle. During an assessment at a local emergency unit an x-ray of the right hip was requested and this was reported as normal (Figure 1).

He was reassured and was discharged but due to ongoing severe pain, the patient contacted his sports physician, who requested a computed tomography scan of the right hip and acetabulum based on suspicion of an underlying fracture.

**Fig. 2:**
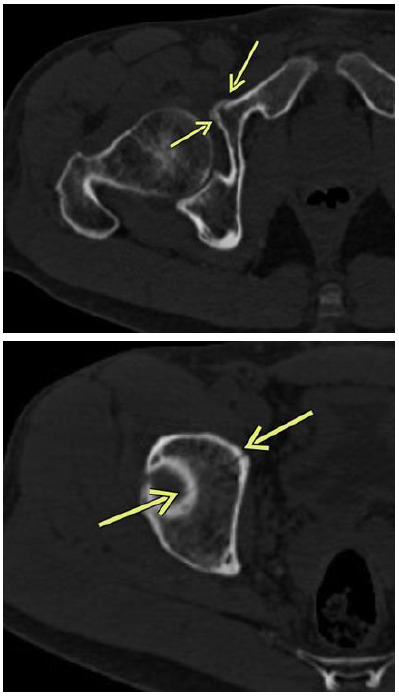
Case 1: Computed tomography scan of the right hip 2 days post injury demonstrating a fracture of the anterior column and extension into the roof of the acetabulum

The scan demonstrated an undisplaced fracture of the anterior column of the right acetabulum, extending into the roof of the right acetabulum. Superiorly, it extended into the right iliac bone and inferiorly extended into the proximal right superior pubic ramus (Figure 2).

The patient was advised to use crutches with no weight bearing for a period of two weeks, followed by partial weight bearing for a further three weeks if pain-free. He was able to commence low-intensity stationary cycling after two weeks and was pain-free and able to resume all normal activities after five weeks.

### Case 2

A 29-year-old competitive female cyclist sustained a fall during a crash in the peloton while competing in a road cycling race. She fell directly onto her right side, with her right hip making contact with the tarmac. She was unable to continue cycling or bear any weight on the affected right leg and was taken directly to the emergency unit. On assessment, an X-ray was requested and was reported as normal.

Due to progressive severe pain and continued inability to weight bear on the right leg, the patient made contact with her sports physician who requested an MRI scan of the right hip and acetabulum on suspicion of a fracture.

**Fig. 3:**
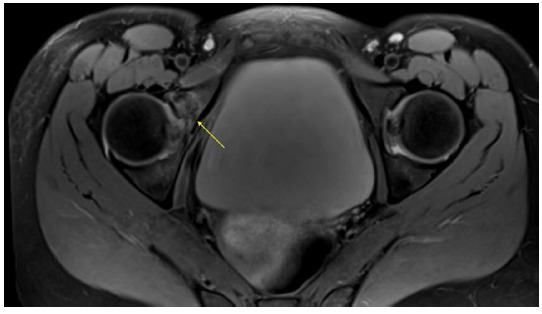
Case 2: MRI scan of the right hip on the day of injury demonstrating a fracture of the anterior column

**Fig. 4:**
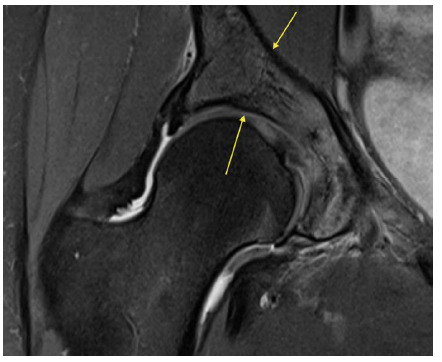
Case 2: MRI scan of the right hip on the day of injury demonstrating fracture of the acetabular roof

This demonstrated a fracture of the anterior column of the right acetabulum (Figure 3) which was undisplaced with related bone marrow and soft tissue oedema. In addition, there was a linear fracture in the coronal plane extending through the acetabular roof (Figure 4) immediately posterior to the midline as well as through the junction of the middle and anterior third of the quadrilateral plate.

The patient was advised to use crutches with no weight bearing for a period of at least three weeks, followed by a period of partial weight bearing for a further three weeks if pain-free. The patient was able to commence low-intensity stationary cycling after three weeks and progressed to gentle low-force cycling outdoors after four weeks. She continued to use one crutch during weight bearing until six weeks at which time she was completely pain-free and able to resume all normal activities.

### Case 3

A 32-year-old professional female cyclist sustained a fall during training and fell directly onto her right side, predominantly onto her hip and shoulder. She was able to continue cycling for approximately 30 minutes but with moderate pain when attempting to produce power with her right leg. She was able to weight bear with minimal pain directly after the fall but her pain increased during that same day and she reported to the emergency unit in the afternoon. Following a clinical assessment, an x-ray was requested and this was reported as normal.

The attending doctor had a high level of suspicion due to the patient’s occupation as a professional cyclist, the nature of her fall and her level of pain, and therefore requested a CT scan.

This demonstrated an undisplaced fracture of the anterior column of the right acetabulum which extended into the iliopubic eminence.

The patient was advised to use crutches and remain non-weight bearing for one week. She was advised to increase weight bearing as the symptoms settled. After two weeks, she was able to walk without assistance.

One week post-injury she commenced low-intensity training on a stationary cycle and progressed from 1h to 2.5h during the first week and to a maximum of 4h at the end of Week two. Three weeks post-injury she was able to return to full training outdoors and has had no further symptoms.

### Case 4

**Fig. 5:**
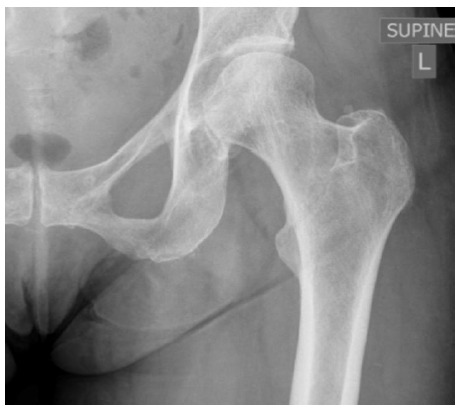
Case 4: X-ray of the left hip one week after the injury

A 68-year-old female recreational cyclist sustained a fall during training and fell onto her left side, directly onto the lateral hip. She was assessed by a physiotherapist and referred for further assessment one week later due to significant pain and disability. An x-ray of the hip and pelvis were assessed as normal (Figure 5) and she was discharged with instructions to undergo MRI scanning if symptoms did not resolve rapidly. The patient subsequently continued to perform activities of daily living while fully weight bearing despite ongoing pain and disability. She returned for a follow-up four weeks later, at which time an MRI scan of the hip and pelvis was performed.

**Fig. 6:**
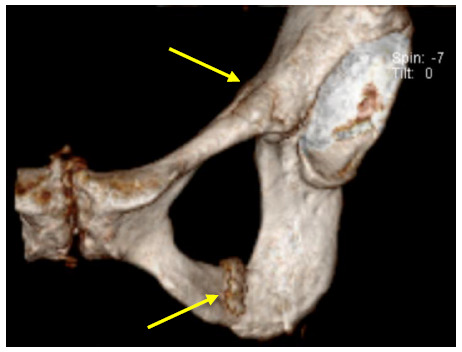
Case 4: 3-D CT scan of the pelvis and left hip four weeks after injury (top arrow showing acetabular fracture, bottom arrow showing ischial ramus fracture and callus formation)

This demonstrated an undisplaced fracture of the anterior column of the right acetabulum as well as an undisplaced fracture of the left ischial ramus, with associated reactive bone oedema, and early callus formation (Figure 6).

She was instructed to non-weight bear for a period of four weeks, with weekly follow-ups. She had some persistent pain, but this gradually settled. After six weeks, with a gradual transition from partial to full weight bearing her symptoms resolved and she was able to weight-bear with no pain and resume stationary cycling as well as progress with her rehabilitation exercises.

## Discussion

We present four cases which we encountered consecutively over a period of 18 months. Each of these cases demonstrated a similar mechanism of injury; direct fall onto the lateral hip when falling during cycling.

Each case was assessed in a trauma centre and three of the four cases were re-assured following plain x-ray imaging. All four cases demonstrated a fracture of the acetabulum on CT or MRI imaging. All four cases sustained a fracture of the anterior column with an extension of the fracture into the roof of the acetabulum (two cases) or anteriorly into the iliopubic eminence (two cases).

Following identification of the fracture each of the cases recovered without complication. In the first three cases with early diagnosis, each was able to ambulate with non-weight bearing for one-three weeks and partial weight bearing for a further two-three weeks after diagnosis. These cases were able to cycle on a stationary cycle between one and three weeks post-injury and all three cases were asymptomatic and returned to full activity between three and six weeks post-injury. One case followed a delayed recovery due to a four week delay in the diagnosis of an acetabular fracture and during which the patient continued to weight bear.

During this same period of time, we did not encounter any patients who presented to our clinic with lateral hip pain following a similar mechanism of injury but where a fracture was not demonstrated. As a result, we can infer that the number of acetabular fractures following falls from bicycles directly onto the lateral hip result in a relatively high number of fractures or that when patients seek further medical attention due to significant pain following this mechanism of injury, there is a high likelihood of fracture. Many of these may be missed due to absence of findings on plain x-ray imaging. Despite the probability of these relatively high rates of bony injury, these patients most likely suffer no sequelae due to the stability of these fractures and the limitation in activity imposed by pain during weight bearing activity.

Clinicians should maintain a high index of suspicion in patients who have direct falls onto the lateral hip and subsequently experience pain with weight bearing activity. Both CT and MRI are excellent modalities to confirm or exclude these fractures.

Patients with confirmed fractures should be managed with non-weight bearing on the affected side for at least one week or until symptoms allow transition to relatively pain free partial weight bearing. Resumption of stationary indoor cycling when symptoms allow may aid recovery by maintaining range of motion and muscle strength.
